# Thymoquinone Inhibits Tumor Growth and Induces Apoptosis in a Breast Cancer Xenograft Mouse Model: The Role of p38 MAPK and ROS

**DOI:** 10.1371/journal.pone.0075356

**Published:** 2013-10-02

**Authors:** Chern Chiuh Woo, Annie Hsu, Alan Prem Kumar, Gautam Sethi, Kwong Huat Benny Tan

**Affiliations:** 1 Department of Pharmacology, Yong Loo Lin School of Medicine, National University of Singapore, Singapore, Singapore; 2 Cancer Science Institute of Singapore, National University of Singapore, Singapore, Singapore; 3 School of Biomedical Sciences, Faculty of Health Sciences, Curtin University, Perth, Western Australia, Australia; 4 Department of Biological Sciences, University of North Texas, Denton, Texas, United States of America; H.Lee Moffitt Cancer Center & Research Institute, United States of America

## Abstract

Due to narrow therapeutic window of cancer therapeutic agents and the development of resistance against these agents, there is a need to discover novel agents to treat breast cancer. The antitumor activities of thymoquinone (TQ), a compound isolated from *Nigella sativa oil*, were investigated in breast carcinoma *in vitro* and *in vivo*. Cell responses after TQ treatment were assessed by using different assays including MTT assay, annexin V-propidium iodide staining, Mitosox staining and Western blot. The antitumor effect was studied by breast tumor xenograft mouse model, and the tumor tissues were examined by histology and immunohistochemistry. The level of anti-oxidant enzymes/molecules in mouse liver tissues was measured by commercial kits. Here, we show that TQ induced p38 phosphorylation and ROS production in breast cancer cells. These inductions were found to be responsible for TQ’s anti-proliferative and pro-apoptotic effects. Moreover, TQ-induced ROS production regulated p38 phosphorylation but not *vice versa*. TQ treatment was found to suppress the tumor growth and this effect was further enhanced by combination with doxorubicin. TQ also inhibited the protein expression of anti-apoptotic genes, such as XIAP, survivin, Bcl-xL and Bcl-2, in breast cancer cells and breast tumor xenograft. Reduced Ki67 and increased TUNEL staining were observed in TQ-treated tumors. TQ was also found to increase the level of catalase, superoxide dismutase and glutathione in mouse liver tissues. Overall, our results demonstrated that the anti-proliferative and pro-apoptotic effects of TQ in breast cancer are mediated through p38 phosphorylation via ROS generation.

## Introduction

In the last decade, numerous papers have reported that thymoquinone (TQ), a compound isolated from *Nigella sativa oil*, was able to suppress a range of carcinomas including breast, liver, prostate and colorectal carcinoma [1]. Many potential targets which TQ regulates for its anticancer activities have been identified including p53 [2,3], p73 [4], STAT3 [5], NF-κB [6], PPAR-γ [7] and reactive oxygen species (ROS) [4,8]. In addition, the combination of TQ with conventional medicine can result in greater anticancer effect, for example in NCI-H460 non-small cell lung cancer cells [9] and U266 multiple myeloma cells [5]. Moreover, TQ can even protect against the toxicity caused by conventional medicine, for example, to ameliorate the nephrotoxicity induced by cisplatin in rodents [10] and the cardiotoxicity of doxorubicin in mice [11]. However, the detailed molecular mechanisms of the antineoplastic effects of TQ are yet to be elucidated, and the potential therapeutic effects of TQ in breast carcinoma are also not clear.

The p38 pathway plays a number of roles including regulation of apoptosis, cell cycle progression, cell growth and differentiation. A number of diseases have been found to be associated with p38 signaling, namely rheumatoid arthritis [12], cardiovascular disease [13] and Parkinson’s disease [14]. Many studies suggest that the p38 pathway may play an important role in cancer as a tumor suppressor. p38 MAPK was shown to up-regulate p16 expression, which in turn inhibits cyclin D1/cdk4 activity [15]. p38 MAPK can stabilize HBP1 protein by phosphorylating it [16], whereby HBP1 can then negatively regulate cell cycle genes, including cyclin D1 and N-myc [17,18]. It had been shown that several chemotherapeutic agents, such as nocodazole, taxol, vincristine and vinblastine, can induce p38 MAPK activation and mitotic cell cycle arrest [19]. The p38 inhibitor was found to reverse nocodazole-induced apoptosis [19]. Moreover, phospho-p38 is almost undetectable in most solid tumors including breast, lung, liver, gastric, renal and ovarian cancers, while this protein is relatively higher expressed in normal organs [20]. Together, these findings explain the potential role of p38 MAPK in anticancer therapy. The agent that can modulate p38 pathway could thus be a solution to tumor malignancy.

ROS are oxygen-containing reactive molecules or ions, which are formed via incomplete one electron reduction of oxygen [21]. Although studies on the effect of ROS in oncology are not fully understood, there are reports suggesting that ROS can promote tumorigenesis through Ras-Raf-MEK-ERK pathway, or suppress tumorigenesis via p38 pathway [21]. It has been reported that ROS, via Ras, can activate ERK1/2, where ERK1/2 plays important roles in tumorigenesis such as cell growth and apoptosis prevention [22,23]. In contrast, ROS was shown to activate p38 MAPK for apoptotic cell death in human cervical cancer cells [24]. The p53/ROS/p38α cascade, whereby p38α can be activated via p53-mediated ROS production, plays an essential role in cisplatin-induced apoptosis in HCT116 colorectal cancer cells [25]. Interestingly, there was a study which suggested that ROS is tumor-promoting, and that p38 MAPK-induced apoptosis is initiated in response to ROS accumulation. This response is believed to play an important role in inhibiting tumor initiation during oxidative stress [26].

Despite the identification of various targets for TQ, the effect of TQ on MAPKs, particularly p38, still remains unexplained. The present work seeks to explain the role of p38 MAPK on the anticancer effects of TQ in breast cancer cells and in the breast tumor xenograft mouse model. We also investigate the role of ROS and its interaction with p38 MAPK. We believe the results will add significant knowledge to the potential use of TQ in breast cancer therapy, in particular its effects on growth inhibition and apoptosis. 

## Materials and Methods

### Chemicals and Antibodies

Trypsin EDTA, trypan blue, thiazolyl blue tetrazolium bromide (MTT), thymoquinone and N-acetylcysteine were purchased from Sigma-Aldrich (St. Louis, MO, USA), while doxorubicin was purchased from Euroasian Chemical Private Ltd. (Mumbai, India). SB203580 was purchased from Promega (WI, USA). RPMI1640 and fetal bovine serum were purchased from Hyclone (Loughborough, UK). Antibiotic-antimycotic mixture was purchased from Gemini Bio-products (West Sacramento, CA, USA). Dimethyl sulfoxide was purchased from MP Biomedicals (Solon, OH, USA). BD matrigel was purchased from BD Biosciences (Franklin Lakes, NJ, USA). Antibodies to Bcl-2, Bcl-xL, Ki67, XIAP, JNK, p-JNK, ERK, p-ERK and PARP were purchased from Santa Cruz Biotechnology (Santa Cruz, CA, USA), while survivin, p38, p-p38 and β-actin were purchased from Cell Signaling (Beverly, MA, USA). Chicken anti-rabbit IgG HRP-conjugated, chicken anti-mouse IgG HRP-conjugated, chicken anti-rabbit IgG TR-conjugated antibodies, p38 siRNA and control siRNA-A were purchased from Santa Cruz Biotechnology.

### Cell lines

MCF-7 and MDA-MB-231 breast cancer cell lines were purchased from ATCC (Manassas, VA, USA). These cell lines were cultured in RPMI1640 medium supplemented with 10% fetal bovine serum and 1% antibiotic-antimycotic. All cell culture were maintained at 37 °C and 5% CO_2_ in a humidified atmosphere.

### 3: (4,5-Dimethylthiazol-2-yl)-2,5-diphenyl-2H-tetrazolium bromide (MTT) assay

The anti-proliferative effect of TQ was assessed by MTT assay. TQ was dissolved in PBS containing 0.5% DMSO for all *in vitro* studies. Briefly, breast cancer cells were seeded (10^4^ cells/well) in a 96-well microtiter plate followed by overnight incubation. After appropriate treatment, 10 µl MTT solution (5 mg/ml) was added to each well for 4 h. The mixture was removed carefully via pipette, and the remaining formazan crystals formed were dissolved by 100 µl DMSO. After 30 mins, the absorbance of each well was read at 570 nm with an absorbance reader (Tecan Infinite M200, Mannedorf, Switzerland).

### Annexin V-propidium iodide analysis

The level of apoptosis of cancer cells was assessed with Annexin V-propidium iodide kit from Miltenyi Biotec (Bergisch Gladbach, Germany). Briefly, breast cancer cells were seeded (2.6 X 10^5^ cells/well) in a 6-well microtiter plate followed by overnight incubation. After appropriate treatment, the cells were trypsinized, washed, and incubated with Annexin V-FITC solution for 15 mins under dark condition. After washing, the cells were analyzed with flow cytometry (CyAn^TM^ ADP from Beckman Coulter, Brea, CA, USA) immediately after propidium iodide solution was added.

### Western blot analysis

The protein expressions of genes of interest in breast cancer cells and breast tumor tissues were measured by Western blot. Briefly, the cells were seeded (2.6 X 10^5^ cells/well) in a 6-well microtiter plate followed by overnight incubation. After appropriate treatment, the cells were trypsinized followed by whole cell lysate extraction. For *in vivo* study, the tumor tissues were homogenized for tissue lysate extraction. Both cell lysate and tissue lysate were centrifuged and the supernatants were collected. After protein estimation with Bio-Rad protein assay (Hercules, CA, USA), a calculated volume of lysate was mixed with laemmli sample buffer, whereby the mixture was resolved by 10% or 12% SDS/PAGE gel and then electroblotted onto a nitrocellulose membrane. The membrane was probed with primary antibody (1:1000) for overnight incubation at 4°C, and then washed and incubated with HRP-conjugated secondary antibody (1:10000) for 1 h at room temperature. The membrane was examined for its chemiluminescence by ECL (GE Healthcare, Little Chalfont, Buckinghamshire, UK). Densitometric analysis of the scanned blots was measured using ImageJ software and the results were expressed as fold change relative to the control after normalization to β-actin.

### ROS measurement

The ROS level of cancer cells was measured by flow cytometry after Mitosox staining (Invitrogen, Carlsbad, CA, USA). Briefly, breast cancer cells were seeded (2.6 X 10^5^ cells/well) in a 6-well microtiter plate followed by overnight incubation. After appropriate treatment, the cells were trypsinized and washed with PBS buffer before mixing with Mitosox-added serum-free medium. The cells were then incubated under dark condition for 15 mins at 37°C before analysis with a flow cytometer (BD LSRII, Franklin Lakes, NJ, USA).

### PathScan® Phospho-p38 MAPK (Thr180/Tyr182) Sandwich ELISA Kit

The p-p38 MAPK level of cancer cells was examined with PathScan® Phospho-p38 MAPK (Thr180/Tyr182) Sandwich ELISA Kit (Cell Signaling, Beverly, MA, USA). The experimental procedures were carried out according to the manufacturer’s protocol. Briefly, breast cancer cells were seeded (2.6 X 10^5^ cells/well) in a 6-well microtiter plate followed by overnight incubation. After appropriate treatment, the cells were lysed followed by centrifugation. The resulting supernatant was added into the wells supplied by the manufacturer. After 4 h incubation at 37°C, the wells were washed with buffer for 4 times. Detection antibody was then added for 1 h at 37°C. The washing step was repeated, followed by incubation for 30 mins with HRP-Linked secondary antibody at 37°C. The washing step was again repeated, followed by incubation for 10 mins with TMB substrate at 37°C. STOP solution was then added into each well for 5 mins. The absorbance was read at 450 nm with an absorbance reader (Tecan Infinite M200, Mannedorf, Switzerland).

### Gene silencing using siRNA

The protein expression of p38/p-p38 was suppressed by siRNA silencing. Briefly, breast cancer cells were seeded (1.7 X 10^5^ cells/well) in a 6-well microtiter plate followed by overnight incubation. The cells were then tranfected with 30 nM of p38 siRNA or control siRNA-A using Oligofectamine tranfection reagent (Invitrogen, Carlsbad, CA, USA) for 6 h according to the manufacturer’s protocol. Serum-added medium was then added for at least 24 h before exposure to appropriate treatment.

### In vivo experiment

Female nude mice (BALB/c OlaHsd-foxn1) were purchased from Biological Resource Centre (BRC, Biopolis, Singapore). The animal protocol was approved by The NUS Institutional Animal Care and Use Committee (IACUC No. 065/11). Upon arrival, the nude mice were kept in individual disposable cages with ventilation, and given food and water ad lib. After acclimatisation over 7 days, each mouse was injected subcutaneously with 10^7^ MDA-MB-231 human breast cancer cells (resuspended in matrigel-added serum free medium) at the right flank region. When the tumor size was about 100 mm^3^ (Volume = ½ X width^2^ X length), the mice were divided into different treatment groups (n=5) as following.

Group 1: Vehicle control saline water (i.p.), 6 days per week.

Group 2: TQ 4 mg/kg (i.p.), 6 days per week.

Group 3: TQ 8 mg/kg (i.p.), 6 days per week.

Group 4: Dox (doxorubicin) 2.5 mg/kg (i.p.), once per week.

Group 5. TQ 4 mg/kg (i.p.), 6 days per week + Dox 2.5 mg/kg (i.p.), once per week.

TQ and doxorubicin were dissolved in saline water containing 5% DMSO. The tumor volume and body weight were measured twice per week. After two weeks of treatment, the mice were euthanized with CO_2_ asphyxiation. Tumor tissues were collected for histological, immunohistochemical and Western blot analysis, while liver tissues were collected for enzymatic assays.

### Hematoxylin and Eosin (H&E) staining

The tumor tissues were placed in 10% neutral buffered formalin solution (Sigma-Aldrich, St. Louis, MO, USA) before being processed and paraffinized. The samples were sectioned and stained with H&E solution (Merck, Germany). The tissue section was examined and photographed with a fluorescence microscope (Olympus BX51, Shinjuku, Japan).

### TUNEL staining

The level of apoptosis of tumor tissues was assessed by TUNEL staining (Promega, WI, USA). The experimental procedures were carried out according to the manufacturer’s protocol. Briefly, the tissue section was deparaffinized before rehydration with decreasing concentrations of ethanol. After washing with 0.85% NaCl and PBS, the tissue section was fixed with 4% formaldehyde for 15 mins. Following washing with PBS, the tissue section was covered with Proteinase K solution for 8-10 mins. After another PBS wash, the tissue section was again fixed with 4% formaldehyde for 5 mins. Following PBS wash, the tissue section was covered with equilibrium buffer for 5-10 mins before addition of TdT reaction mixture. After incubation under dark condition for 1 h, the tissue section was incubated with SSC solution for 15 mins, followed by a final PBS wash. After DAPI counterstain, the tissue section was examined and photographed with a fluorescence microscope (Olympus BX51, Shinjuku, Japan). Average number of fluorescence dots of three images from each treatment group was calculated.

### Ki67 immunohistochemistry

The tissue section was deparaffinized before undergoing antigen retrieval step with citrate buffer. The tissue section was next blocked with 2% fetal bovine serum for 20-30 mins, and then incubated with rabbit anti-human Ki67 antibody (1:200) for 1 h at room temperature. After rinsing with PBS, the tissue section was incubated with chicken anti-rabbit IgG TR-conjugated antibody (1:500) for 1 h under dark condition. Following DAPI counterstain, the tissue section was examined and photographed with a fluorescence microscope (Olympus BX51, Shinjuku, Japan). Average number of fluorescence dots of three images from each treatment group was calculated.

### Catalase assay

The catalase level in mouse liver tissues was measured using the catalase assay kit from Cayman Chemical (Ann Arbor, Michigan, USA). The experimental procedures were carried out according to the manufacturer’s protocol. Briefly, the liver tissues were homogenized in cold buffer (50 mM potassium phosphate, 1 mM EDTA, pH 7). The supernatant was collected after centrifugation. The sample was mixed with diluted assay buffer and methanol in a 96-well microtiter plate. The reaction was initiated by adding diluted hydrogen peroxide for 20 mins with constant shaking. Diluted potassium hydroxide was then added followed by catalase purpald. The plate was incubated immediately for 10 mins with constant shaking. Catalase potassium periodate was then added followed by 5 mins incubation with constant shaking. The absorbance was then read at 540 nm with an absorbance reader (Tecan Infinite M200, Mannedorf, Switzerland).

### Superoxide dismutase (SOD) assay

The SOD level in mouse liver tissues was measured using the SOD assay kit from Cayman Chemical (Ann Arbor, Michigan, USA). The experimental procedures were carried out according to the manufacturer’s protocol. Briefly, the liver tissues were homogenized in HEPES buffer (20 mM HEPES buffer, 1 mM EGTA, 210 mM mannitol, 70 mM sucrose, pH 7.2). The supernatant was collected after centrifugation. The sample was added to diluted radical detector in a 96-well microtiter plate. The reaction was initiated by adding diluted xanthine oxidase. The plate was incubated immediately for 20 mins with constant shaking. The absorbance was then read at 450 nm with an absorbance reader (Tecan infinite M200, Mannedorf, Switzerland).

### Glutathione assay

The glutathione level in mouse liver tissues was measured using the glutathione assay kit from Cayman Chemical (Ann Arbor, Michigan, USA). The experimental procedures were carried out according to the manufacturer’s protocol. Briefly, the liver tissues were homogenized in cold buffer (50 mM phosphate, 1 mM EDTA, pH 6-7). The supernatant was collected after centrifugation. The sample was first deproteinated by triethanolamine. The sample was added to assay cocktail in a 96-well microtiter plate. The plate was incubated immediately for 25 mins under dark condition with constant shaking. The absorbance was then read at 405 nm with an absorbance reader (Tecan Infinite M200, Mannedorf, Switzerland).

### Statistical analysis

Statistical analysis was performed by one way analysis of variance (ANOVA). A p-value of less than 0.05 was considered to be statistically significant. 

## Results

### MAPKs protein phosphorylation after TQ treatment

**Figure 1:**
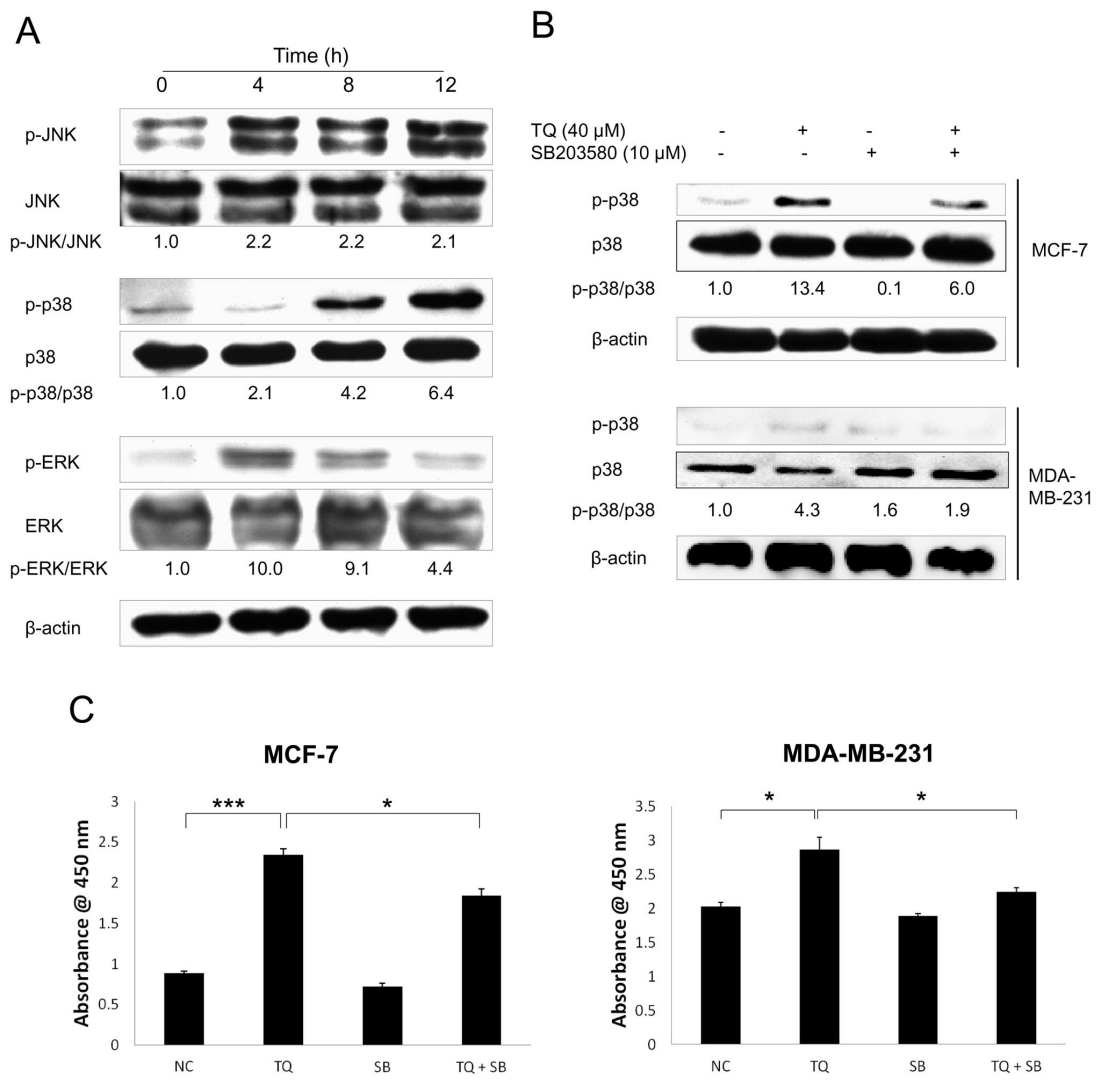
TQ induces MAPKs protein phosphorylation, particularly p38, in breast cancer cells. (A) Western blot analysis showed the protein phosphorylation of MAPKs by TQ. MCF-7 cells were treated with 40 µM TQ for various time periods ranging up to 12 h. Data are representative of at least three independent experiments. (B) Western blot analysis showed the effect of SB203580 on TQ-induced p38 phosphorylation. MCF-7 and MDA-MB-231 cells were pre-treated with 10 µM SB203580 for 1 h before exposure to 40 µM TQ for 12 h. Data are representative of at least three independent experiments. (C) The effect of SB203580 on TQ-induced p-p38 level was shown by PathScan® phospho-p38 MAPK (Thr180/Tyr182) sandwich ELISA kit as described under Materials and Methods section. MCF-7 and MDA-MB-231 cells were pre-treated with 10 µM SB203580 for 1 h before exposure to 40 µM TQ for 12 h. Values are means ± S.E.M. of at least three independent experiments. * p<0.05, *** p<0.001.

We first determined whether TQ can induce any effect in MAPKs protein phosphorylation in breast cancer cells. Western blot analysis demonstrated that TQ significantly up-regulated the phosphorylation of various MAPKs in MCF-7 cells (Figure 1A). The increase of JNK and p38 protein phosphorylation was found to be maximal at 12 h. On the other hand, the increase of ERK protein phosphorylation peaked at 4 h and gradually decreased till 12 h.

### The specific p38 inhibitor (SB203580) abrogates TQ-induced p38 phosphorylation

Having determined the potential effect of TQ on p38 MAPK, we further investigated the specificity of this effect on both MCF-7 and MDA-MB-231 breast cancer cell lines. Both cell lines were pre-treated with 10 µM SB203580 followed by TQ treatment. In both cell lines, TQ was found to induce the phosphorylation of p38, and this induction was reversed by SB203580 treatment (Figure 1B). In addition to western blot, a p38 MAPK ELISA kit (as described in Materials and Methods) was also used to measure the p-p38 level in TQ-treated cells. We found that TQ significantly increased the p-p38 level in both cell lines after exposure to 40 µM TQ for 12 h (Figure 1C). This increase was also significantly reversed by SB203580 treatment.

### The involvement of p38 MAPK in TQ-induced anti-proliferative and pro-apoptotic effects

**Figure 2:**
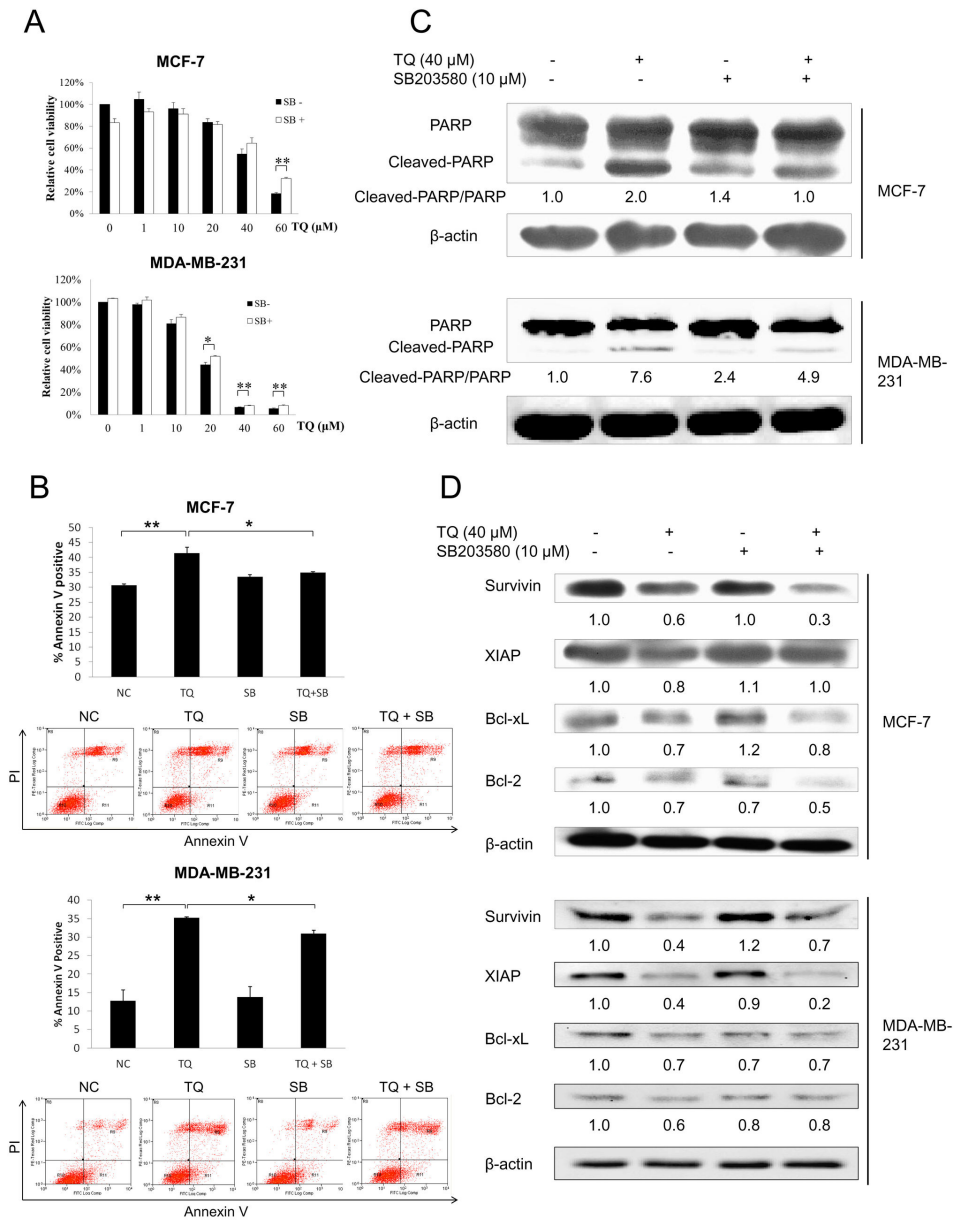
The role of p38 MAPK on TQ-induced anti-proliferative and pro-apoptotic effects in breast cancer cells. (A) MTT assay results showed the effect of SB203580 on TQ-induced anti-proliferative effect in MCF-7 and MDA-MB-231 cells. The cells were pre-treated with 10 µM SB203580 for 1 h before exposure to increasing doses of TQ for 24 h. Values are means ± S.E.M. of at least three independent experiments. * p<0.05, ** p<0.01. (B) Annexin V assay results showed the effect of SB203580 on TQ-induced pro-apoptotic effect in MCF-7 and MDA-MB-231 cells. The cells were pre-treated with 10 µM SB203580 for 1 h before exposure to 40 µM TQ (50 µM in MCF-7 cells) for 12 h. Data are representative of at least three independent experiments. Values are means ± S.E.M. of at least three independent experiments. * p<0.05, ** p<0.01. (C) Western blot analysis showed the effect of SB203580 on TQ-induced PARP protein cleavage in MCF-7 and MDA-MB-231 cells. The cells were pre-treated with 10 µM SB203580 for 1 h before exposure to 40 µM TQ for 12 h. Data are representative of at least three independent experiments. (D) Western blot analysis showed the effect of TQ and SB203580 on the protein expression of various anti-apoptotic genes in MCF-7 and MDA-MB-231 cells. The cells were pre-treated with 10 µM SB203580 for 1 h before exposure to 40 µM TQ for 12 h. Data are representative of at least three independent experiments.

MCF-7 and MDA-MB-231 cells were treated with increasing doses of TQ for 24 h with or without SB203580 treatment. The results from MTT assay indicated that SB203580 treatment significantly reversed the anti-proliferative effect of TQ in both cell lines, at least partially (Figure 2A). We also examined whether the level of p38 phosphorylation interfered with TQ-induced apoptosis. We found that SB203580 treatment significantly reversed TQ-induced increased percentage of Annexin V positive cells (Figure 2B). As shown in Figure 2C, the cleaved-PARP protein in both cell lines was increased after TQ treatment, and this increase was reversed when the cells were pre-treated with SB203580. We also investigated the protein expression of various anti-apoptotic/pro-survival genes such as survivin, XIAP, Bcl-xL and Bcl-2. In both cell lines, we found that TQ suppressed the protein expression of these genes, however, these suppressions were not all reversed by SB203580 treatment (Figure 2D). We found that the decrease of XIAP in MCF-7 cells by TQ could be reversed by SB203580 treatment. On the other hand, the decrease of survivin and Bcl-2 by TQ was reversed by SB203580 treatment in MDA-MB-231 cells.

### N-acetylcysteine (NAC) prevents TQ-induced ROS production

**Figure 3:**
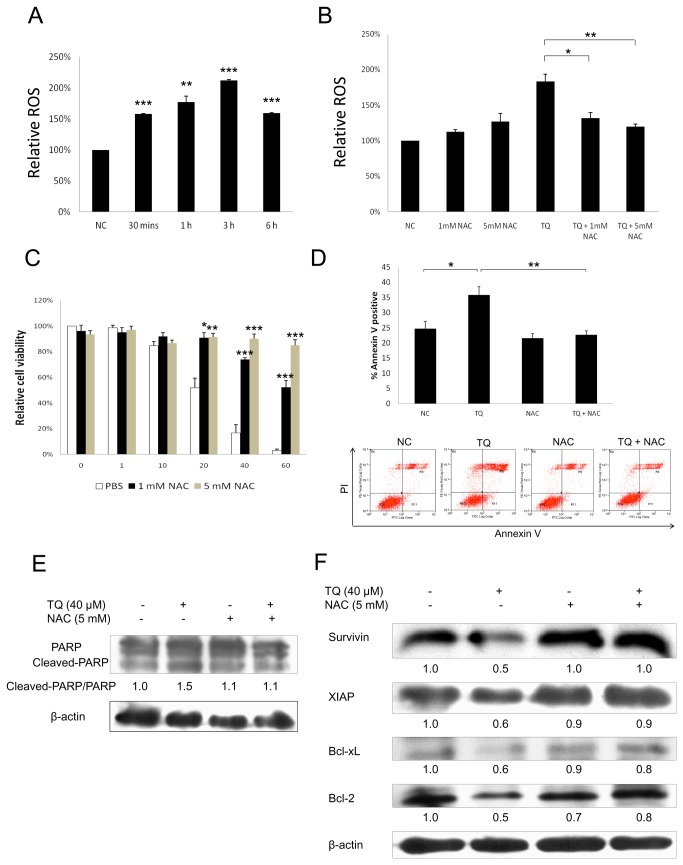
The role of ROS in the anti-proliferative and pro-apoptotic effects induced by TQ in breast cancer cells. (A) Mitosox assay results showed the effect of TQ on ROS production in MCF-7 cells. The cells were treated with 40 µM TQ for various time periods ranging up to 6 h. Values are means ± S.E.M. of at least three independent experiments. ** p<0.01, *** p<0.001 vs. negative control. (B) Mitosox assay results showed the effect of NAC on TQ-induced ROS production. MCF-7 cells were pre-treated with 1 or 5 mM NAC for 2 h before exposure to 40 µM TQ for 3 h. Values are means ± S.E.M. of at least three independent experiments. * p<0.05, ** p<0.01. (C) MTT assay results showed the effect of NAC on TQ-induced anti-proliferative effect in MCF-7 cells. The cells were pre-treated with 1 or 5 mM NAC for 2 h before exposure to increasing doses of TQ for 24 h. Values are means ± S.E.M. of at least three independent experiments. * p<0.05, ** p<0.01, *** p<0.001 vs. PBS control. (D) Annexin V assay results showed the effect of NAC on TQ-induced pro-apoptotic effect in MCF-7 cells. The cells were pre-treated with 5 mM NAC for 2 h before exposure to 50 µM TQ for 12 h. Data are representative of at least three independent experiments. Values are means ± S.E.M. of at least three independent experiments. * p<0.05, ** p<0.01. (E) Western blot analysis showed the effect of NAC on PARP protein cleavage in MCF-7 cells. The cells were pre-treated with 5 mM NAC for 2 h before exposure to 40 µM TQ for 12 h. Data are representative of at least three independent experiments. (F) Western blot analysis showed the effect of TQ and NAC on the protein expression of various anti-apoptotic genes. MCF-7 cells were pre-treated with 5 mM NAC for 2 h before exposure to 40 µM TQ for 12 h. Data are representative of at least three independent experiments.

There are papers reporting that TQ mediates ROS production as a mechanism to induce apoptosis and growth inhibition in various cancer cells [8,27,28] except breast cancer. In this study, we demonstrated the effect of TQ on ROS production in breast cancer cells, and its effect on cell proliferation and apoptosis. MCF-7 cells were treated with 40 µM TQ for various time periods ranging up to 6 h. As shown in Figure 3A, TQ significantly induced ROS production as early as 30 mins, and this induction was time-dependent up to 3 h after TQ treatment. TQ-induced ROS production was reversed by pre-2 h treatment with NAC, a strong antioxidant (Figure 3B).

### The role of ROS in TQ-induced anti-proliferative and pro-apoptotic effects

We also investigated whether ROS level can interfere with TQ-induced growth inhibition. We found that the anti-proliferative effect of TQ in MCF-7 cells was reversed by NAC in a dose-dependent manner (Figure 3C). Next, we pre-treated MCF-7 cells with 5 mM NAC for 2 h before exposure to TQ for 12 h. Both TQ-induced increased percentage of Annexin V positive cells (Figure 3D) and cleavage of PARP protein (Figure 3E) were reversed by NAC treatment, indicating apoptosis reversal through ROS reduction. We also examined whether the level of ROS inter-relates with the protein expression of various anti-apoptotic/pro-survival genes. We found that the decrease of survivin, XIAP, Bcl-xL and Bcl-2 protein expression by TQ were all reversed by NAC treatment (Figure 3F).

### p38 MAPK gene silencing reversed TQ-induced apoptosis

**Figure 4:**
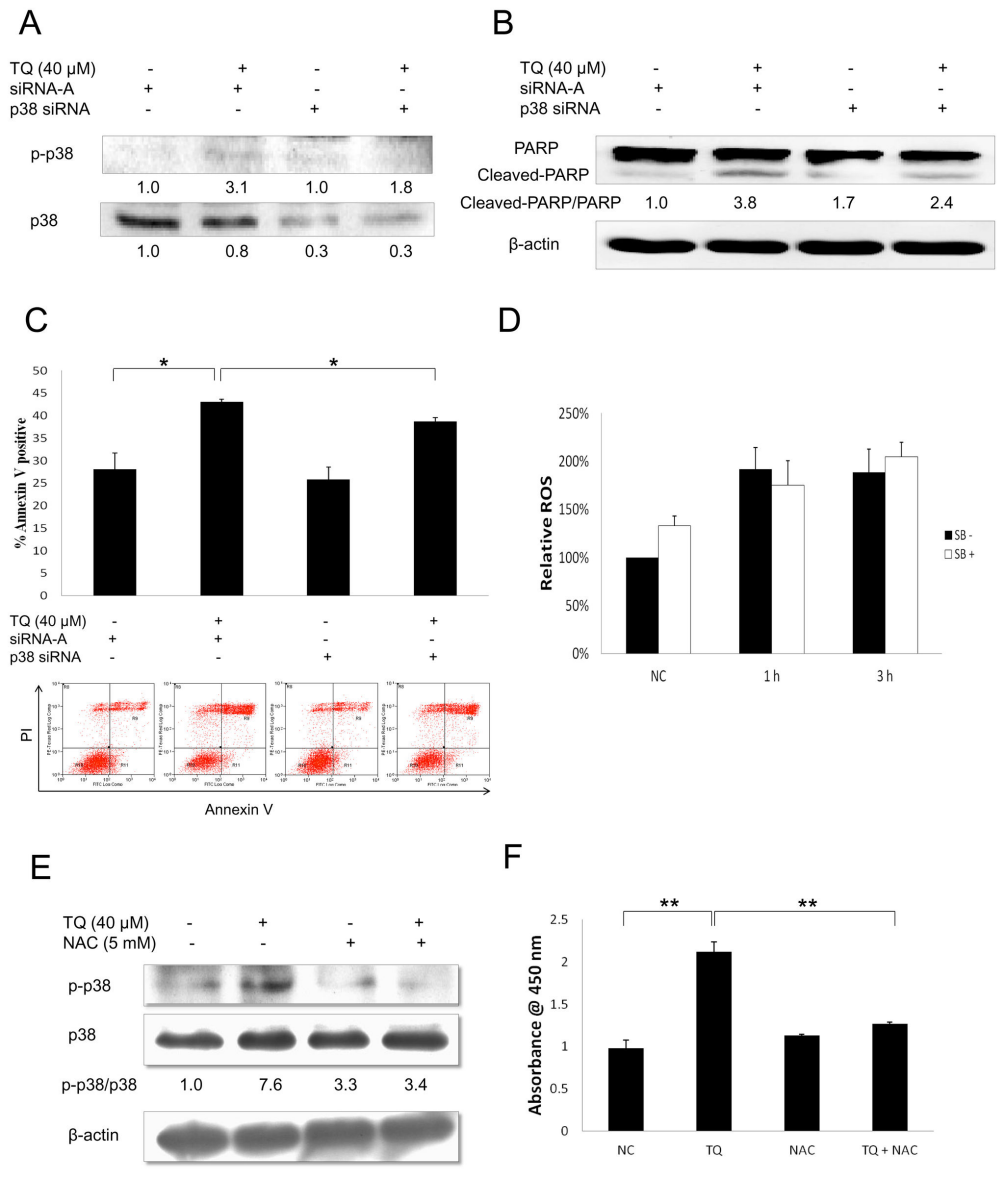
Effect of p38 siRNA gene silencing on the apoptotic effect of TQ, and investigation on the relationship between p38 and ROS. (A) Western blot analysis showed the effect of p38 siRNA gene silencing on the protein expressions of p-p38 and p38. MCF-7 cells were transfected with 30 nM p38 siRNA for 6 h followed by at least 24 h incubation with serum-added medium. The cells were then treated with 40 µM TQ for 12 h. Data are representative of at least three independent experiments. (B) Western blot analysis showed the effect of p38 siRNA gene silencing on TQ-induced PARP protein cleavage in MCF-7 cells. The cells were transfected with 30 nM p38 siRNA for 6 h followed by at least 24 h incubation with serum-added medium. The cells were treated with 40 µM TQ for 12 h. Data are representative of at least three independent experiments. (C) Annexin V assay results showed the effect of p38 siRNA gene silencing on TQ-induced increased percentage of Annexin V positive cells. The cells were transfected with 30 nM p38 siRNA for 6 h followed by at least 24 h incubation with serum-added medium. The cells were treated with 50 µM TQ for 12 h. Data are representative of at least three independent experiments. Values are means ± S.E.M. of at least three independent experiments. * p<0.05. (D) Mitosox assay results showed the effect of SB203580 on TQ-induced ROS level. MCF-7 cells were pre-treated with 10 µM SB203580 for 1 h before exposure to 40 µM TQ for 1 or 3 h. Values are means ± S.E.M. of at least three independent experiments. (E) Western blot analysis showed the effect of NAC on TQ-induced p38 phosphorylation. MCF-7 cells were pre-treated with 5 mM NAC for 2 h before exposure to 40 µM TQ for 12 h. Data are representative of at least three independent experiments. (F) The effect of NAC on TQ-induced p-p38 level was shown by PathScan® phospho-p38 MAPK (Thr180/Tyr182) sandwich ELISA kit. MCF-7 cells were pre-treated with 5 mM NAC for 2 h before exposure to 40 µM TQ for 12 h. Values are means ± S.E.M. of at least three independent experiments. ** p<0.01.

As shown in Figure 4A, both p-p38 and p38 protein expressions were reduced with p38 siRNA transfection. Moreover, we found that TQ-induced PARP-cleavage (Figure 4B) and increased percentage of Annexin V positive cells (Figure 4C) were both partially reversed by p38 siRNA transfection, which confirmed the role of p-p38 in TQ-induced apoptosis.

### ROS regulates p38 phosphorylation

Since TQ was shown to affect ROS and p38 pathways, we investigated the relationship between ROS and p38 MAPK. MCF-7 cells were pre-treated with 10 µM SB203580 for 1 h before exposure to 40 µM TQ for 1 h or 3 h. We found that SB203580 treatment did not make any significant changes on TQ-induced ROS level (Figure 4D). This indicates that p38 phosphorylation level did not affect the level of ROS. Next, we pre-treated MCF-7 cells with 5 mM NAC for 2 h before exposure to 40 µM TQ for 12 h. Through Western blot analysis, we found that NAC treatment reversed TQ-induced p38 phosphorylation (Figure 4E). Furthermore, the results from the p38 MAPK ELISA kit also showed that NAC treatment could significantly reverse TQ-induced p-p38 level (Figure 4F). These results indicate that TQ-induced ROS regulates the phosphorylation of p38 in MCF-7 cells.

### TQ inhibits breast tumor growth in nude mice

**Figure 5:**
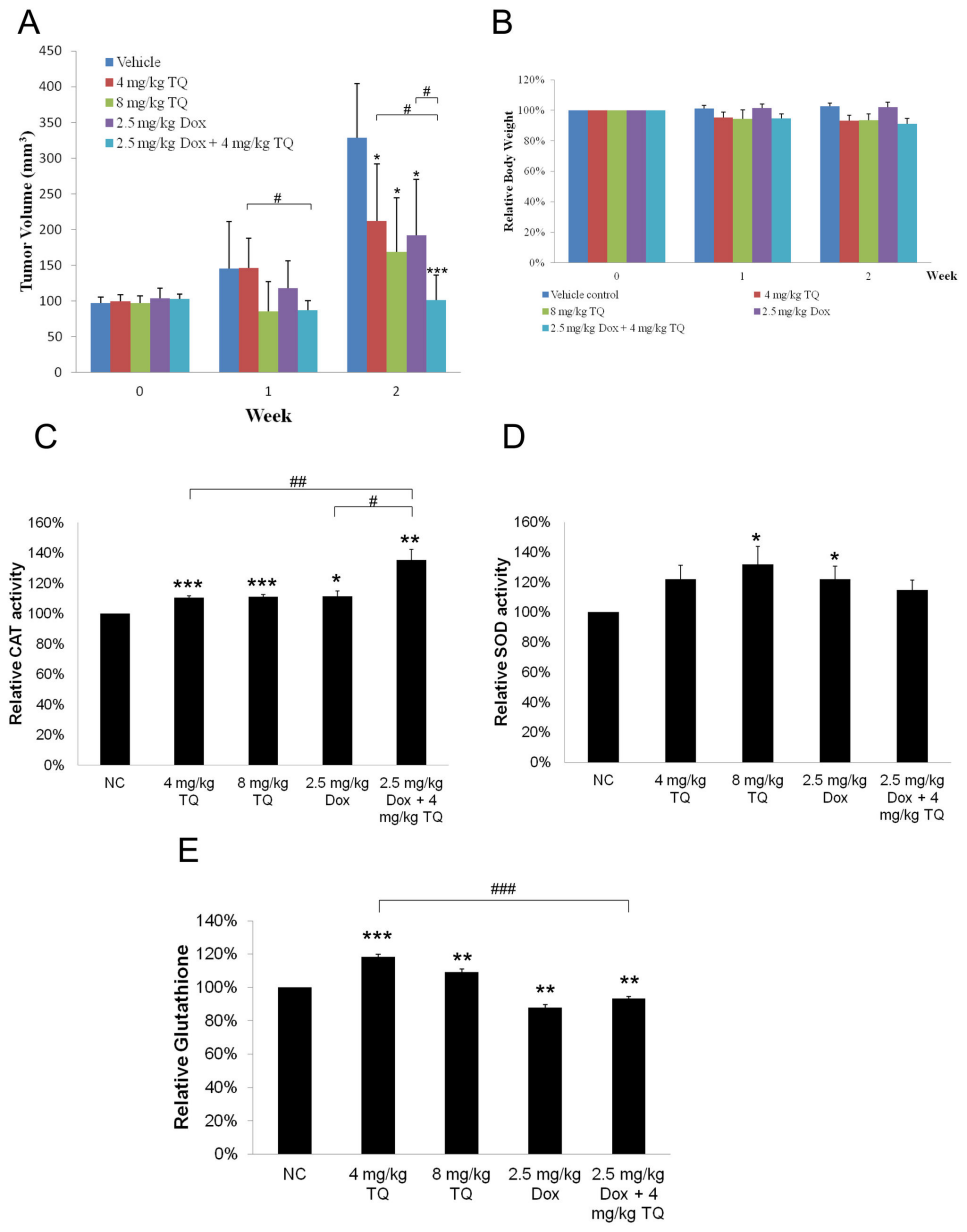
TQ suppresses breast tumor growth in nude mice, and increases levels of anti-oxidant enzymes/molecules in liver tissues. (A) Tumor volume of each treatment group during study period. 10^7^ MDA-MB-231 cells were injected subcutaneously into the right flank region of each nude mouse. When tumor volume reached 100 mm^3^, the mice were divided into five treatment groups, each n=5. Group I: saline water (i.p., 6 days per week), group II: 4 mg/kg TQ (i.p., 6 days per week), group III: 8 mg/kg TQ (i.p., 6 days per week), group IV: 2.5 mg/kg Dox (i.p., once per week), and group V: 2.5 mg/kg Dox (i.p., once per week) + 4 mg/kg TQ (i.p., 6 days per week). Tumor volume was measured with Vernier calipers and calculated from the formula, V = (width^2^ X length)/2. Values are means ± S.D. of each group. * p<0.05, *** p<0.001 compared to the control group. # p<0.05 is comparison in between the indicated pair. (B) Mouse body weight relative to the starting measurement. Values are means ± S.D. of each group. (C-E) Enzymatic assays results showed the effect of TQ on the levels of catalase (C), SOD (D) and glutathione (E) in mouse liver tissues. Mouse liver tissues were collected for enzymatic assays as described under Materials and Methods. Values are means ± S.E.M. of at least three independent experiments. * p<0.05, ** p<0.01, *** p<0.001 compared to the vehicle group. # p<0.05, # # p<0.01, # # # p<0.001 are comparisons in between the indicated pair.

MDA-MB-231 breast cancer cells were injected subcutaneously into the right flank region of female nude mice to develop breast tumor xenograft. As shown in Figure 5A, the tumor volume of vehicle group was increased aggressively after 2 weeks (from about 100 mm^3^ to about 330 mm^3^). Treatment with 4 mg/kg TQ, 8 mg/kg TQ and 2.5 mg/kg Dox significantly slowed the tumor growth, though they did not completely eliminate the tumors or return to the start level. The combined treatment (4 mg/kg TQ + 2.5 mg/kg Dox) slowed tumor growth more significantly than either agent alone. Although the combined treatment did not completely eliminate the tumor, it could maintain the tumor growth at the start level throughout the 2 weeks period of treatment. We also found that TQ treatment slightly reduced mouse body weight although no visible adverse effect was observed (Figure 5B). Less than 9% body weight reduction was observed for all TQ-treated groups including 4 mg/kg TQ, 8 mg/kg TQ and the combined treatment group.

### The level of anti-oxidant enzymes/molecules in mouse liver tissues

Since TQ was found to produce ROS in cancer cells, we also investigated its effect in *in vivo* model by measuring antioxidant enzymes/molecules in mouse liver tissues. We found that the catalase level was significantly increased in TQ-treated groups compared to the vehicle group (Figure 5C). The catalase level was significantly higher in the combined treatment group compared to the either agent alone. On the other hand, SOD level was significantly higher in the 8 mg/kg TQ and 2.5 mg/kg Dox groups compared to the vehicle group (Figure 5D). Though not statistically significant, 4 mg/kg TQ and the combined treatment groups had higher mean SOD levels compared to the vehicle group. We also found that glutathione level was significantly higher in TQ alone groups (Figure 5E). Interestingly, the glutathione level in the 2.5 mg/kg Dox group was lower compared to the vehicle group. Glutathione level was lower in the combined treatment group compared to the vehicle group but higher than in the 2.5 mg/kg Dox group.

### Histology, immunohistochemistry and Western blot analysis of tumor tissues

**Figure 6:**
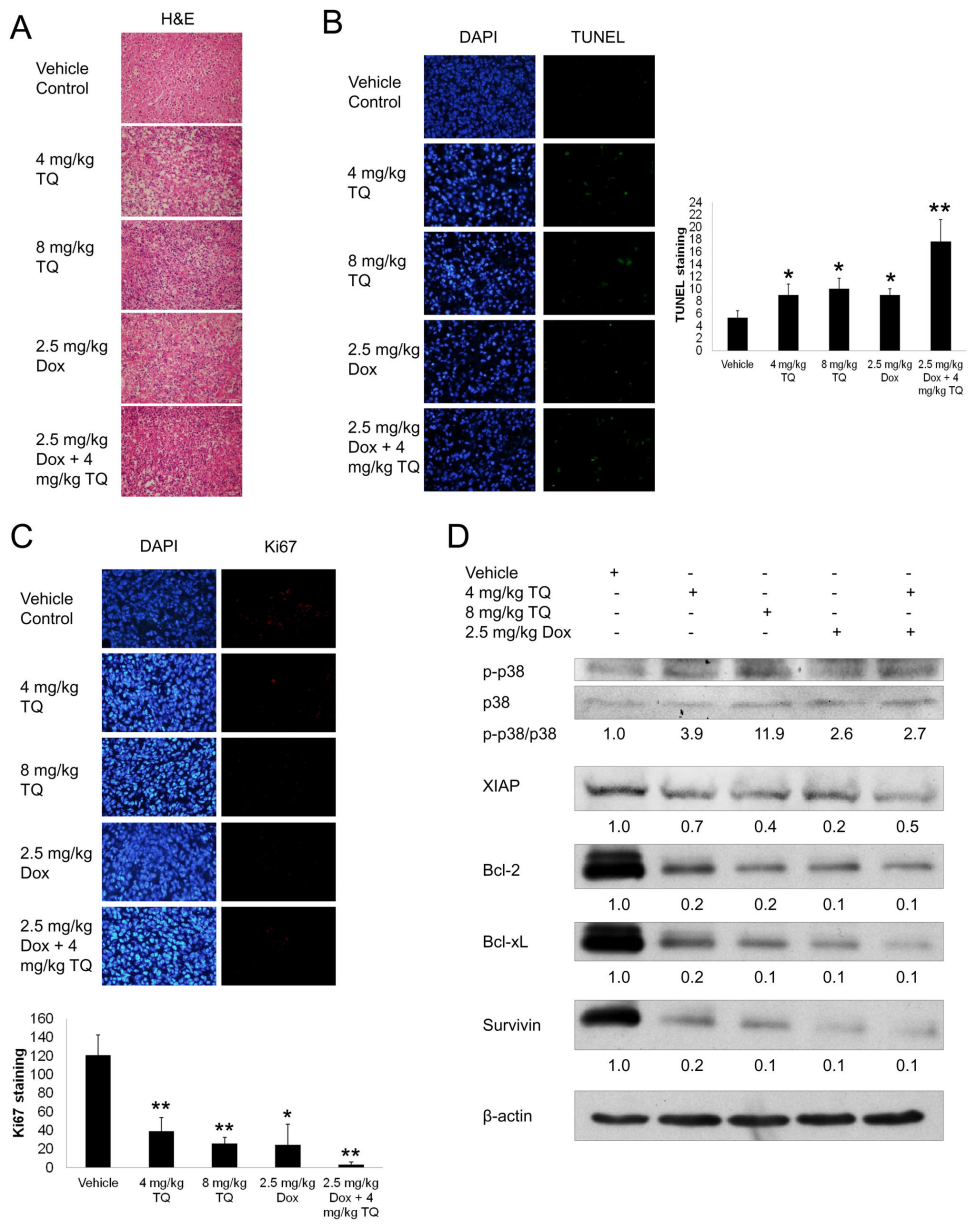
TQ induces apoptosis in breast tumor xenograft with down-regulation of anti-apoptotic proteins. (A) H&E staining of the tumor tissues. (B) TUNEL staining of the tumor tissues. The graph showed the average number of fluorescence dots of images from each treatment group. Values are means ± S.D. of three photographs. * p<0.05, ** p<0.01 compared to the vehicle group. (C) Ki67 immunohistochemical staining of the tumor tissues. The graph showed the average number of fluorescence dots of images from each treatment group. Values are means ± S.D. of three photographs. * p<0.05, ** p<0.01 compared to the vehicle group. (D) Western blot analysis showed the effect of drug treatments on the protein expression of various anti-apoptotic genes. Data are representative of at least three independent experiments.

The tumor tissues were subjected to H&E staining for structure analysis. The vehicle group displayed high grade tumor with irregular cell arrangement (Figure 6A). In contrast, in the drug treatment groups, alterations in cell architecture were observed as characterized by an increase in cell debris and a decrease in stroma. In addition, TUNEL staining was carried out to study the level of apoptosis of tumor tissues. By calculating the number of green fluorescent dots relative to the vehicle group, we observed about 50% higher TUNEL staining in 4 mg/kg TQ, 8 mg/kg TQ and 2.5 mg/kg Dox groups, and about 150% higher staining in the combined treatment group, compared to the vehicle group (Figure 6B). Furthermore, immunohistochemistry of the tumor tissues targeting Ki67 protein, a cellular marker for proliferation showed that the Ki67 level significantly reduced in the drug treatment groups, compared to the vehicle group, with the lowest level in the combined treatment group (Figure 6C). Western blot analysis of homogenized tumor tissues showed that p-p38 protein expression was increased in the TQ-treated groups, but not in the Dox alone group, compared to vehicle (Figure 6D). The protein expression of anti-apoptotic/pro-survival genes, such as survivin, XIAP, Bcl-xL and Bcl-2, were decreased in the drug treatment groups, compared to the vehicle group (Figure 6D). 

## Discussion

In this study, we explored the potential effects of TQ on ROS and p38 pathways both *in vitro* and *in vivo*. This is the first report to suggest that TQ induced ROS production, which, in turn, resulted in p38 phosphorylation, contributing to TQ’s anti-proliferative and pro-apoptotic effects in breast cancer. In the xenograft mouse model, we showed the ability of TQ to suppress breast tumor growth, and the combined treatment with doxorubicin to cause significantly higher suppression. Moreover, TQ was found to increase p-p38 protein expression in tumor tissues, with down-regulation of XIAP, survivin, Bcl-xL and Bcl-2 anti-apoptotic gene products. TQ treatment also increased catalase, SOD and glutathione levels in mouse liver tissues.

Recent developments have raised a question on the role of ROS, whether it protects against or promotes oxidative stress [29]. Accumulating evidence suggests that ROS found in various chronic diseases might not be the result of disease damage, but instead, could be the response of host to the disease. As such, researchers start using pro-oxidant agents to increase oxidative stress in cancer cells as a strategy to target resistant tumor cells [30]. For example, TQ-induced ROS production was found to down-regulate Akt in primary effusion lymphoma cells [28]. In addition, TQ was shown to induce ROS-mediated ERK and JNK phosphorylation in human colon cancer cells [8]. These results have led us to suggest that TQ’s anticancer effects may be mediated upstream at ROS. Many studies have identified potential targets of TQ such as p53 [2,3], p73 [4], STAT3 [5], NF-κB [6] and PPAR-γ [7]. One may raise a question on whether these targets are also regulated by ROS. A recent review by Maillet and Pervaiz (2012) explained how ROS production regulates p53 activity [31]. In addition, STAT3 activation was found to be mediated via ROS in pulmonary epithelial cells [32] and B lymphocytes [33]. However, this kind of relationship is not always one way, for example, p53 can act as upstream regulator of ROS production by binding to promoters such as GPX and PIGs [31]. Therefore, the role of ROS in TQ-induced apoptosis requires further comprehensive investigation with well-designed models.

Numerous studies have suggested that p38 MAPK can act as a tumor suppressor by negatively regulating cell cycle progression, p53 activation and oncogene-induced premature senescence [34]. It was shown that p38α regulates the proliferation and differentiation of lung stem and progenitor cells, and inactivation of this pathway can lead to K-Ras(G12V)-induced tumorigenesis [35]. Furthermore, p38α protein expression was found to be approximately 3 times lower in human lung tumors than in human normal lung tissues [35]. In the present study, we show that p38 MAPK plays an important role in TQ’s anti-proliferative and pro-apoptotic effects. In contrast, the study by El-Najjar et al. (2010) described the ability of TQ to increase the phosphorylation of JNK and ERK, but not p38, in human colon cancer cells via ROS [8]. Interestingly, the phosphorylation of JNK and ERK was found to serve as a survival mechanism in TQ-induced cell death, and the inhibition of these MAPKs can potentiate TQ-induced apoptosis [8]. Nevertheless, both El-Najjar’s study and this study have explained the role of ROS as an upstream mediator of phosphorylation of MAPKs, and this fact should further be explored for its role in cancer therapeutics.

The antitumor effects of TQ have been shown in other types of carcinoma including lung [9,36], pancreas [37], prostate [38], gastric [39] and colon [40]. We found that TQ and doxorubicin in combination suppressed tumor growth more significantly than either agent alone. A similar finding was also reported for the combination of TQ and 5-fluorouracil [39], and TQ and cisplatin [9]. Moreover, TQ in combination with gemcitabine or oxaliplatin produced greater antitumor effect than either agent alone in the pancreatic tumor xenograft mouse model [37]. These results strongly suggest the possible use of TQ as a complementary agent to potentiate the antitumor effect of conventional anticancer drugs.

We found that TQ increased the levels of catalase, SOD and glutathione in liver tissues of mouse xenograft model. These enzymes/molecules are generally known for their involvement in cellular anti-oxidative activities. However, we are not certain whether the increase of these enzymes/molecules was due to TQ induction or the response of cellular defense mechanisms against TQ-induced ROS production. TQ has been shown to reverse the decrease of glutathione peroxidase, glutathione-S-transferase, catalase, and reduced glutathione in kidney and liver tissues of streptozotocin nicotinamide-induced diabetic rat [41]. TQ-induced glutathione level in female Lewis rats with experimental allergic encephalomyelitis is believed to improve the condition of the disease [42]. In contrast, there was a study reporting that TQ treatment did not change the level of reduced glutathione or glutathione-S-transferase in liver and kidney tissues of normal mice [43]. As such, the nature of TQ as anti-oxidant or pro-oxidant in different models has to be further explored.

In conclusion, our study provides evidence for the mechanism of action of TQ in suppressing human breast carcinoma in both *in vitro* and *in vivo* models. We demonstrated that the anti-proliferative and pro-apoptotic effects of TQ are mediated through its induction effect on p38 and ROS signaling. Our results also indicate the anti-tumor effects of TQ in breast tumor xenograft mice and its ability to potentiate the antitumor effect of doxorubicin. TQ serves as a promising anticancer agent and further studies may provide important leads for its clinical application.

## References

[1] Woo CC, Kumar AP, Sethi G, Tan KH (2012) Thymoquinone: potential cure for inflammatory disorders and cancer. Biochem Pharmacol 83: 443-451. doi:10.1016/j.bcp.2011.09.029. PubMed: 22005518. 22005518

[2] Gali-Muhtasib H, Diab-Assaf M, Boltze C, Al-Hmaira J, Hartig R et al. (2004) Thymoquinone extracted from black seed triggers apoptotic cell death in human colorectal cancer cells via a p53-dependent mechanism. Int J Oncol 25: 857-866. PubMed: 15375533. 15375533

[3] Gali-Muhtasib H, Kuester D, Mawrin C, Bajbouj K, Diestel A et al. (2008) Thymoquinone triggers inactivation of the stress response pathway sensor CHEK1 and contributes to apoptosis in colorectal cancer cells. Cancer Res 68: 5609-5618. doi:10.1158/0008-5472.CAN-08-0884. PubMed: 18632613. 18632613

[4] Alhosin M, Abusnina A, Achour M, Sharif T, Muller C et al. (2010) Induction of apoptosis by thymoquinone in lymphoblastic leukemia Jurkat cells is mediated by a p73-dependent pathway which targets the epigenetic integrator UHRF1. Biochem Pharmacol 79: 1251-1260. doi:10.1016/j.bcp.2009.12.015. PubMed: 20026309. 20026309

[5] Li F, Rajendran P, Sethi G (2010) Thymoquinone inhibits proliferation, induces apoptosis and chemosensitizes human multiple myeloma cells through suppression of signal transducer and activator of transcription 3 activation pathway. Br J Pharmacol 161: 541-554. doi:10.1111/j.1476-5381.2010.00874.x. PubMed: 20880395. 20880395 PMC2990154

[6] Sethi G, Ahn KS, Aggarwal BB (2008) Targeting nuclear factor-kappa B activation pathway by thymoquinone: role in suppression of antiapoptotic gene products and enhancement of apoptosis. Mol Cancer Res 6: 1059-1070. doi:10.1158/1541-7786.MCR-07-2088. PubMed: 18567808. 18567808

[7] Woo CC, Loo SY, Gee V, Yap CW, Sethi G et al. (2011) Anticancer activity of thymoquinone in breast cancer cells: possible involvement of PPAR-γ pathway. Biochem Pharmacol 82: 464-475. doi:10.1016/j.bcp.2011.05.030. PubMed: 21679698. 21679698

[8] El-Najjar N, Chatila M, Moukadem H, Vuorela H, Ocker M et al. (2010) Reactive oxygen species mediate thymoquinone-induced apoptosis and activate ERK and JNK signaling. Apoptosis 15: 183-195. doi:10.1007/s10495-009-0421-z. PubMed: 19882352. 19882352

[9] Jafri SH, Glass J, Shi R, Zhang S, Prince M et al. (2010) Thymoquinone and cisplatin as a therapeutic combination in lung cancer: In vitro and in vivo. J Exp Clin Cancer Res 29: 87. doi:10.1186/1756-9966-29-87. PubMed: 20594324. 20594324 PMC2909169

[10] Badary OA, Nagi MN, al-Shabanah OA, al-Sawaf HA, al-Sohaibani MO et al. (1997) Thymoquinone ameliorates the nephrotoxicity induced by cisplatin in rodents and potentiates its antitumor activity. Can J Physiol Pharmacol 75: 1356-1361. doi:10.1139/y97-169. PubMed: 9534946. 9534946

[11] Al-Shabanah OA, Badary OA, Nagi MN, al-Gharably NM, al-Rikabi AC et al. (1998) Thymoquinone protects against doxorubicin-induced cardiotoxicity without compromising its antitumor activity. J Exp Clin Cancer Res 17: 193-198. PubMed: 9700580. 9700580

[12] Pargellis C, Regan J (2003) Inhibitors of p38 mitogen-activated protein kinase for the treatment of rheumatoid arthritis. Curr Opin Investig Drugs 4: 566–571. PubMed: 12833650. 12833650

[13] Behr TM, Berova M, Doe CP, Ju H, Angermann CE et al. (2003) p38 mitogen-activated protein kinase inhibitors for the treatment of chronic cardiovascular disease. Curr Opin Investig Drugs 4: 1059–1064. PubMed: 14582449. 14582449

[14] Wilms H, Rosenstiel P, Sievers J, Deuschl G, Zecca L et al. (2003) Activation of microglia by human neuromelanin is NF-kappaB dependent and involves p38 mitogen-activated protein kinase: Implications for Parkinson’s disease. FASEB J 17: 500–502. PubMed: 12631585. 12631585 10.1096/fj.02-0314fje

[15] Molnár A, Theodoras AM, Zon LI, Kyriakis JM (1997) Cdc42Hs, but not Rac1, inhibits serum-stimulated cell cycle progression at G1/S through a mechanism requiring p38/RK. J Biol Chem 272: 13229–13235. doi:10.1074/jbc.272.20.13229. PubMed: 9148940. 9148940

[16] Xiu M, Kim J, Sampson E, Huang CY, Davis RJ et al. (2003) The transcriptional repressor HBP1 is a target of the p38 mitogen-activated protein kinase pathway in cell cycle regulation. Mol Cell Biol 23: 8890–8901. doi:10.1128/MCB.23.23.8890-8901.2003. PubMed: 14612426. 14612426 PMC262665

[17] Sampson EM, Haque ZK, Ku MC, Tevosian SG, Albanese C et al. (2001) Negative regulation of the Wnt-beta-catenin pathway by the transcriptional repressor HBP1. EMBO J 20: 4500–4511. doi:10.1093/emboj/20.16.4500. PubMed: 11500377. 11500377 PMC125566

[18] Tevosian SG, Shih HH, Mendelson KG, Sheppard KA, Paulson KE et al. (1997) HBP1: A HMG box transcriptional repressor that is targeted by the retinoblastoma family. Genes Dev 11: 383–396. doi:10.1101/gad.11.3.383. PubMed: 9030690. 9030690

[19] Deacon K, Mistry P, Chernoff J, Blank JL, Patel R (2003) p38 mitogen-activated protein kinase mediates cell death and p21-activated kinase mediates cell survival during chemotherapeutic drug-induced mitotic arrest. Mol Cell Biol 14: 2071–2087. doi:10.1091/mbc.E02-10-0653. PubMed: 12802076. PMC165098 12802076

[20] Liao Y, Hung MC (2003) Regulation of the activity of p38 mitogen-activated protein kinase by Akt in cancer and adenoviral protein E1A-mediated sensitization to apoptosis. Mol Cell Biol 23: 6836–6848. doi:10.1128/MCB.23.19.6836-6848.2003. PubMed: 12972603. 12972603 PMC193925

[21] Pan JS, Hong MZ, Ren JL (2009) Reactive oxygen species: A double-edged sword in oncogenesis. World J Gastroenterol 15: 1702-1707. doi:10.3748/wjg.15.1702. PubMed: 19360913. 19360913 PMC2668775

[22] Abe J, Okuda M, Huang Q, Yoshizumi M, Berk BC (2000) Reactive oxygen species activate p90 ribosomal S6 kinase via Fyn and Ras. J Biol Chem 275: 1739-1748. doi:10.1074/jbc.275.3.1739. PubMed: 10636870. 10636870

[23] Aikawa R, Komuro I, Yamazaki T, Zou Y, Kudoh S et al. (1997) Oxidative stress activates extracellular signal-regulated kinases through Src and Ras in cultured cardiac myocytes of neonatal rats. J Clin Invest 100: 1813-1821. doi:10.1172/JCI119709. PubMed: 9312182. 9312182 PMC508367

[24] Kang YH, Lee SJ (2008) The role of p38 MAPK and JNK in Arsenic trioxide-induced mitochondrial cell death in human cervical cancer cells. J Cell Physiol 217: 23-33. doi:10.1002/jcp.21470. PubMed: 18412143. 18412143

[25] Bragado P, Armesilla A, Silva A, Porras A (2007) Apoptosis by cisplatin requires p53 mediated p38alpha MAPK activation through ROS generation. Apoptosis 12: 1733-1742. doi:10.1007/s10495-007-0082-8. PubMed: 17505786. 17505786

[26] Dolado I, Swat A, Ajenjo N, De Vita G, Cuadrado A et al. (2007) p38alpha MAP kinase as a sensor of reactive oxygen species in tumorigenesis. Cancer Cell 11: 191-205. doi:10.1016/j.ccr.2006.12.013. PubMed: 17292829. 17292829

[27] Koka PS, Mondal D, Schultz M, Abdel-Mageed AB, Agrawal KC (2010) Studies on molecular mechanisms of growth inhibitory effects of thymoquinone against prostate cancer cells: role of reactive oxygen species. Exp Biol Med (Maywood) 235: 751-760. doi:10.1258/ebm.2010.009369. PubMed: 20511679. 20511679

[28] Hussain AR, Ahmed M, Ahmed S, Manogaran P, Platanias LC et al. (2011) Thymoquinone suppresses growth and induces apoptosis via generation of reactive oxygen species in primary effusion lymphoma. Free Radic Biol Med 50: 978-987. doi:10.1016/j.freeradbiomed.2010.12.034. PubMed: 21215312. 21215312

[29] Naviaux RK (2012) Oxidative shielding or oxidative stress? J Pharmacol Exp Ther 342: 608-618. doi:10.1124/jpet.112.192120. PubMed: 22700427. 22700427

[30] Martin-Cordero C, Leon-Gonzalez AJ, Calderon-Montano JM, Burgos-Moron E, Lopez-Lazaro M (2012) Pro-oxidant natural products as anticancer agents. Curr Drug Targets 13: 1006-1028. doi:10.2174/138945012802009044. PubMed: 22594470. 22594470

[31] Maillet A, Pervaiz S (2012) Redox regulation of p53, redox effectors regulated by p53: a subtle balance. Antioxid Redox Signal 16: 1285-1294. doi:10.1089/ars.2011.4434. PubMed: 22117613. 22117613

[32] Choi SY, Lim JW, Shimizu T, Kuwano K, Kim JM et al. (2012) Reactive oxygen species mediate Jak2/Stat3 activation and IL-8 expression in pulmonary epithelial cells stimulated with lipid-associated membrane proteins from Mycoplasma pneumoniae. Inflamm Res 61: 493-501. doi:10.1007/s00011-012-0437-7. PubMed: 22270622. 22270622

[33] Nadeau PJ, Roy A, Gervais-St-Amour C, Marcotte MÈ, Dussault N et al. (2012) Modulation of CD40-activated B lymphocytes by N-acetylcysteine involves decreased phosphorylation of STAT3. Mol Immunol 49: 582-592. doi:10.1016/j.molimm.2011.10.007. PubMed: 22078209. 22078209

[34] Bulavin DV, Fornace AJ Jr (2004) p38 MAP kinase’s emerging role as a tumor suppressor. Adv Cancer Res 92: 95-118. doi:10.1016/S0065-230X(04)92005-2. PubMed: 15530558. 15530558

[35] Ventura JJ, Tenbaum S, Perdiguero E, Huth M, Guerra C et al. (2007) p38alpha MAP kinase is essential in lung stem and progenitor cell proliferation and differentiation. Nat Genet 39: 750-758. doi:10.1038/ng2037. PubMed: 17468755. 17468755

[36] Attoub S, Sperandio O, Raza H, Arafat K, Al-Salam; S, et al. (2012) Thymoquinone as an anticancer agent: evidence from inhibition of cancer cells viability and invasion in vitro and tumor growth in vivo. Fundam Clin Pharmacol doi: 10.1111/j.1472-8206.2012.01056.x.

[37] Banerjee S, Kaseb AO, Wang Z, Kong D, Mohammad M et al. (2009) Antitumor activity of gemcitabine and oxaliplatin is augmented by thymoquinone in pancreatic cancer. Cancer Res 69: 5575-5583. doi:10.1158/0008-5472.CAN-08-4235. PubMed: 19549912. 19549912

[38] Kaseb AO, Chinnakannu K, Chen D, Sivanandam A, Tejwani S et al. (2007) Androgen receptor and E2F-1 targeted thymoquinone therapy for hormone-refractory prostate cancer. Cancer Res 67: 7782-7788. doi:10.1158/0008-5472.CAN-07-1483. PubMed: 17699783. 17699783

[39] Lei X, Lv X, Liu M, Yang Z, Ji M et al. (2012) Thymoquinone inhibits growth and augments 5-fluorouracil-induced apoptosis in gastric cancer cells both in vitro and in vivo. Biochem Biophys Res Commun 417: 864-868. doi:10.1016/j.bbrc.2011.12.063. PubMed: 22206670. 22206670

[40] Gali-Muhtasib H, Ocker M, Kuester D, Krueger S, El-Hajj Z et al. (2008) Thymoquinone reduces mouse colon tumor cell invasion and inhibits tumor growth in murine colon cancer models. J Cell Mol Med 12: 330-342. PubMed: 18366456. 18366456 10.1111/j.1582-4934.2007.00095.x PMC3823493

[41] Sankaranarayanan C, Pari L (2011) Thymoquinone ameliorates chemical induced oxidative stress and β-cell damage in experimental hyperglycemic rats. Chem Biol Interact 190: 148-154. doi:10.1016/j.cbi.2011.02.029. PubMed: 21382363. 21382363

[42] Mohamed A, Shoker A, Bendjelloul F, Mare A, Alzrigh M et al. (2003) Improvement of experimental allergic encephalomyelitis (EAE) by thymoquinone; an oxidative stress inhibitor Biomed Sci Instrum 39: 440-445. PubMed: 12724933 12724933

[43] Mansour MA, Nagi MN, El-Khatib AS, Al-Bekairi AM (2002) Effects of thymoquinone on antioxidant enzyme activities, lipid peroxidation and DT-diaphorase in different tissues of mice: a possible mechanism of action. Cell Biochem Funct 20: 143-151. doi:10.1002/cbf.968. PubMed: 11979510. 11979510

